# Relating movement behaviours and non-motor characteristics in people with Parkinson's disease: A compositional data analysis approach

**DOI:** 10.1177/1877718X251384816

**Published:** 2025-10-07

**Authors:** Kristina Larsson, Hanna Johansson, Daniel Peterson, Jenny Sedhed, Breiffni Leavy

**Affiliations:** 1 Department of Neurobiology, Care Sciences and Society, Division of Physiotherapy, Karolinska Institutet, Stockholm, Sweden; 2 Department of Community Medicine and Rehabilitation, Physiotherapy, Umeå University, Umeå, Sweden; 3 Research and development unit, Stockholm Sjukhem Foundation, Stockholm, Sweden; 4 College of Health Solutions, Arizona State University, Phoenix, Arizona, USA

**Keywords:** Parkinson's disease, physical activity, non-motor characteristics, compositional data analysis

## Abstract

**Background:**

People with Parkinson's disease (PwPD) have unhealthier movement behaviours (less moderate-to-vigorous physical activity (MVPA) and more sedentary behaviour (SB)), than healthy older adults. Associations across movement patterns and non-motor characteristics are poorly understood.

**Objectives:**

To investigate associations between relative time spent in MVPA, light-intensity physical activities (LIPA) and SB, and non-motor characteristics among PwPD, and to investigate theoretical changes in non-motor characteristics when time in different movement behaviours is reallocated.

**Methods:**

Baseline data from 119 participants in the STEPS randomised controlled trial was used. Movement behaviours were measured by ActiGraph GT3X accelerometers. Compositional data analysis assessed relative time in MVPA, LIPA and SB. Linear regression assessed associations between MVPA, LIPA and SB and self-reported anxiety and depression (HADS), executive function (TMT IV), self-efficacy for exercise (S-ESES) and activities-specific balance confidence (ABC). Isotemporal substitution modelling investigated theoretical changes in outcomes when time in MVPA, LIPA and SB were reallocated.

**Results:**

Better executive function was associated with more relative time in MVPA and less in LIPA. Higher exercise-self-efficacy was associated with more relative time in MVPA and less in SB. Better balance confidence related to more relative time in MVPA. Reallocating time showed that losing 20 min MVPA had a worse theoretical impact for these outcomes than the benefit of gaining 20 min.

**Conclusions:**

The observed relationships between MVPA and executive function, balance confidence, and exercise-self-efficacy suggests particular importance of maintaining MVPA in PwPD. These findings can be utilized clinically by communicating the importance of maintaining time in MVPA among PwPD.

## Plain language title

How is movement linked to non-motor characteristics in people with Parkinson's disease?

**Plain language summary:**

People living with Parkinson's disease tend to move less and spend more time sitting still compared to healthy older adults. While we know that the extent to which people move affects their health, we don’t fully understand how different types of movement are connected to other aspects of daily life in Parkinson's disease such as mood, thinking skills, balance confidence, or belief in the ability to exercise.

This study explored how different kinds of daily movement — including moderate-to-vigorous physical activity (like brisk walking), light-intensity activity (like slow walking or household chores), and sedentary behaviour (like sitting or lying down while awake) — are linked to non-movement-related characteristics in people with Parkinson's. We also looked at what might happen if people spent more or less time in these different movement categories.

To do this, data was collected from people with Parkinson's who took part in a research project called the STEPS study. Participants wore a small activity monitor for one week to track their daily movement. They also answered questionnaires about anxiety and depression, cognitive function and belief in their capacity to exercise and maintain balance.

The results showed that people who spent more in moderate-to-vigorous physical activity tended to have better cognitive function, believed more in their capacity to exercise, and had greater balance confidence. Interestingly, findings also suggest that a reduction in time spent in higher-intensity activity (loss of 20 min moderate-to-vigorous activity) might have a worse effect on health outcomes than the benefit of adding 20 extra minutes.

## Introduction

The adoption of healthy movement behaviour—being sufficiently physically active and spending less time in sedentary behaviour—is important for the management of symptoms in Parkinson's disease (PD). There is systematic evidence for the beneficial effects of healthy movement behaviour, in conjunction with pharmaceutical treatment, on a wide range of PD motor symptoms, such as gait, balance, musculoskeletal strength and aerobic capacity.^1–3^ In addition to motor symptoms, people with PD exhibit a diverse range of non-motor characteristics, such as mood disorders and cognitive impairment. While these symptoms are known to impact quality of life, they are often under-acknowledged and undertreated.^4^ Additionally, relationships between movement behaviour and PD non-motor characteristics are not fully understood. Evidence suggests that healthy movement behaviours relate to decreased levels of depression^4–6^ and anxiety^7^ and improved cognition.^4,8^ Among people with PD, physical factors can affect movement behaviour as well as psychological factors. Specifically, self-efficacy for exercise, defined as an individual's belief in their capacity to overcome personal, social, and environmental barriers to physical activity,^9^ is recognised as a key factor influencing movement behaviour in PD.^10^ An additional influential factor is a person's perceived confidence in their balance.^11^ However, current research on the impact of psychological factors on movement behaviours remains limited in PD.

In previously published research, specific movement behaviours have generally been examined in isolation, independent of their separate parts. These parts can be defined as light-intensity physical activities (LIPA), moderate-to-vigorous physical activities (MVPA), and sedentary behaviour (SB).^12^ However, MVPA, LIPA and SB are interdependent behaviours, such that if time in one behaviour increases, time in one (or several) of the other behaviours must decrease. If, for example, the time a person with PD spends in SB is reallocated to time spent in MVPA, this shift could incur positive effects on health and quality of life.^13^ Compositional Data Analysis (CoDA) is a novel method used to analyse movement behaviours as relative to each other, as opposed to the more traditional methods where each behaviour is analysed separately in terms of absolute time.^12,14^ The use of CoDA together with compositional isotemporal substitution analysis allows us to theoretically test the effect of time reallocation across different movement behaviours on specific health outcomes.^15^ To our knowledge, this approach has not yet been used in the published literature to characterise movement behaviour in the PD population.

People with PD have more unhealthy movement behaviours than healthy older adults,^16–18^ with those in mild to moderate stages of PD spending 75–80% of their waking hours in SB and only 2–3% in MVPA.^16,17^ Motivating and supporting people with PD to engage in healthy movement behaviours is often a main goal of rehabilitation and advice should be person-centred.^19,20^ Precision-based exercise prescription requires a detailed understanding of specific movement behaviours in PD, and how they relate to disease characteristics. However, there is insufficient evidence for the relative benefits of different exercise intensities on health in PD, due to a lack of adequately powered studies.^1^ In particular, knowledge concerning the relationships between specific movement behaviours and non-motor characteristics is lacking. Therefore, the aim of this study was to investigate associations between relative time in the movement behaviours MVPA, LIPA and SB, and non-motor characteristics in people with PD, and to investigate the theoretical changes which occur in non-motor characteristics when time in different movement behaviours is reallocated. We hypothesise that more relative time in MVPA and less relative time spent in SB will be associated with higher levels of self-efficacy for exercise and cognition, and lower levels of depression and anxiety. Furthermore, we hypothesise that reallocation of time from SB to MVPA will be associated with similar changes.

## Materials and methods

### Study design and population

This study is based on the baseline measurements of community-dwelling people with mild to moderate PD who participated in the Support for home Training using Ehealth in Parkinsons diseaSe (STEPS), a double blind randomized controlled trial (RCT). The baseline measurements were collected between 2022 and 2024 within primary health care at one clinical site. Inclusion criteria for the STEPS trial were a diagnosis of PD (as documented by the presence of ICD-10 code G20 in the individual's medical records), Hoehn & Yahr at stage 1–3, Montreal Cognitive Assessment score ≥ 21 points, age ≥ 50 years and being able to walk independently indoors for six minutes without a walking aid. The exclusion criteria were, no internet connection in the home, pre-existing orthopedic or neurological diseases affecting gait, visual or hearing impairments impeding intervention delivery and > 2 falls one month prior to inclusion. All tests were performed during the ON-phase of the participants medication. After the baseline measurement, participants in STEPS were randomised to either the intervention (a ten-week motor-cognitive eHealth training with cognitive behavioural components to increase physical activity levels) or an active control group (a ten-week paper-based home exercise program). The primary outcome in the RCT was walking capacity assessed by the six-minute walk test. More details about the RCT can be found in the study protocol.^21^ Prior to participation, all participants signed a written informed consent form. STEPS was carried out according to the Declaration of Helsinki, approved by the Swedish Ethical Review Authority (Dnr: 2022-02979-01 and 2023-0071702) and registered on Clinicaltrials.gov in August 2022 (NCT 05510739).

At the baseline measurement, demographic data was collected via interview-administered questionnaires as well as clinical performance tests. The next day, participants started to wear an ActiGraph GT3X accelerometer to enable physical activity data collection prior to commencement of the intervention.

### Measurement of non-motor characteristics

Non-motor characteristics were collected using patient-reported questionnaires and clinical tests. Depression and anxiety were measured with the Hospital Anxiety and Depression Scale (HADS), which is a 14-item questionnaire with two subscales for depression and anxiety respectively, with seven items for each subscale. It was based on a 4-point Likert response scale ranging from 0 to 3, resulting in a total score ranged from 0 to 21 for the depression scale, and from 0 to 21 for the anxiety scale. HADS is a valid measure for people with PD.^22^

Executive function was assessed using the Trail Making Test (TMT) condition IV from Delis Kaplan Executive Function System (D-KEFS).^23^ Participants were instructed to connect both numbers and letters in an ascending and alternating order, by drawing a line from one point to the next as quickly as possible (e.g., 1-a-2-b-3-c-4-d, etc.). The outcome was time taken to complete the test, where faster times indicate greater executive function. If not completed within 240 s, the test was interrupted.

Self-efficacy for exercise was measured with the Swedish version of the exercise self-efficacy scale (S-ESES).^24^ This was based on a 4-point Likert response scale (1 = not at all true; 2 = rarely true; 3 = moderately true; and 4 = always true) and addressed ten statements concerning confidence while performing physical activity and exercise. A sum score of all sub-items ranging from 10 (low self-efficacy) to 40 (high self-efficacy) was used as the outcome. S-ESES is reliable among people with neurological disease.^24^

Activities-specific balance confidence was measured with the ABC scale^25^ which assesses balance confidence while performing a variety of balance challenging daily activities. The mean score of all 16 scale items is used, whereby each activity is scored from 0% (not confident at all) to 100% (completely confident). The Swedish version of the ABC scale is a valid and reliable measure in people with PD.^26^

### Measurement of movement behaviours

Movement behaviour data, involving time spent in MVPA, LIPA and SB were collected with ActiGraph GT3X (Acti-Graph, Pensacola, FL, US) accelerometers. Participants were instructed to wear the accelerometer, secured to an elastic belt and positioned on their right hip, during waking hours over a period of seven consecutive days. Additionally, participants logged their daily wear time in a diary. The diaries were used to verify wear time and the number of valid days. The accelerations were sampled at 30 Hz. The raw accelerometer data was processed by using the GGIR package version 3.2-6 in R statistical software.^27^ The GGIR package auto-calibrated the raw triaxial accelerometer signals and computed the Euclidean Norm Minus One (ENMO) metric (i.e., gravity-corrected vector magnitude units). ENMO values were expressed in milli-gravitational units (mg). GGIR considered non-wear time as periods longer than 90 min with no recorded data. The data was categorised based on cut points for elderly people as SB (ENMO <15 mg), LIPA (ENMO 15–68 mg) and MVPA (ENMO ≥69 mg).^28^ To be included in the analysis, participants needed to have at least three valid days of accelerometer data with at least 10 h of wear time per day.

### Statistical analysis

The accelerometer data was analysed with Compositional Data Analysis (CoDA).^12^ First, compositional means of time spent in MVPA, LIPA and SB were calculated by creating the geometric mean and summarising the behaviours to 100%. The daily time for each participant was expressed as a set of two isometric log-ratio (ilr) coordinates, including all relative information about the three compositional parts, as exemplified below for MVPA.


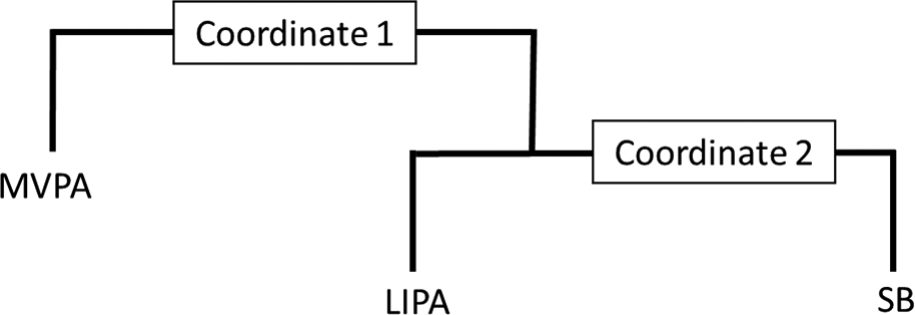

$${\mathrm{ilr}}_{1}=\sqrt{\frac{2}{3}}\ln\frac{\mathrm{MVPA}}{\sqrt[{2}]{\mathrm{LIPA}\;x\;\mathrm{SB}}}$$

$${\mathrm{ilr}}_{2}=\sqrt{\frac{1}{2}}\ln\frac{\mathrm{SB}}{\sqrt[{1}]{\mathrm{LIPA}}}$$

Linear regression analysis investigated the associations between each movement behaviour (in relation to the others) and each of the non-motor characteristics. The set of two ilr coordinates for each movement behaviour was used as explanatory variables, with the first ilr coordinate containing all the relative information about this behaviour, relative to the geometric mean of the remaining ones. Thus, one linear regression model was conducted for each outcome (depression, anxiety, executive functions, self-efficacy for exercise, and balance confidence) with three different sets of ilr coordinates (two ilr coordinates for each movement behaviour). The regression models were conducted stepwise for each outcome; 1: crude model with the outcome and movement behaviours, 2: adding covariates age, sex, and disease duration. These covariates were chosen as they have the potential to relate to both our independent and dependent variables. In particular, the choice of disease duration as a surrogate for disease severity, as other metrics (such as MDS-UPDRS) were not available in this cohort.

When significant associations between any movement behaviour and non-motor characteristics was observed, the effect on the outcome was further explored by reallocating time in 5-min intervals from the behaviour to and from time in another behaviour by using compositional isotemporal substitution analysis.^15^ The composition was adjusted to sum up to the mean wear time. For each outcome, a linear regression model first predicted the outcome value based on the mean time-use composition. Then, a linear regression model predicted the outcome for a composition where time was reallocated from one behaviour (e.g., MVPA) to an alternative behaviour (e.g., SB), keeping the third behaviour (e.g., LIPA) constant (one-for-one reallocation). The difference in the outcomes was estimated as the difference between the outcome value predicted from the reallocated composition and the outcome value predicted from the mean composition.

Assumptions for linear regression and normal distribution of included variables were assessed using histograms, QQ-plots, and residual plots.^29^ For all analyses, a p-value of ≤0.05 was considered as statistically significant. All analysis was performed in R using the Compositions package.^30^ An analysis plan was uploaded at Open Science Framework (OSF) on August 7, 2024 (https://osf.io/u86qm/), before the analysis was performed.

## Results

In total, 119 participants were included in the analysis. Table 1 describes the baseline characteristics of the sample. Overall, mean age (± SD) was 69.8 ± 7.4 years and 50.4% of participants were female.

**Table 1.** Descriptive characteristics of the total sample.

Variable	Total sample (n = 119)
Female	60 (50.4)
Age, years	69.8 (7.4)
Years since PD diagnosis	6.9 (5.2)
Mini-BESTest (0–28)	22.6 (3.1)
10-meter walk test	8.0
Hoehn and Yahr stage:	
1	2 (1.7)
2	75 (63.0)
3	42 (35.3)
Movement behaviours	
Absolute time:	
MVPA, min	32.9 (22.5)
LIPA, min	127.0 (64.8)
SB, min	635.6 (90.4)
Relative time:	
MVPA, min	23.4
LIPA, min	118.8
SB, min	653.3
Isometric log-ratio coordinate number 1:	
MVPA, ilr_1_	−2.0 (0.9)
LIPA, ilr_1_	−0.03 (0.4)
SB, ilr_1_	2.1 (0.6)
Accelerometer wear time, min	795.5 (63.5)
HADS Depression (0–21)	3.0 (2.3)
HADS Anxiety (0–21)	4.8 (3.2)
TMT IV, seconds (0–240)	125.6 (54.6)
S-ESES (0–40)	27.9 (6.7)
ABC (0–100)	81.4 (15.6)

Mini-BESTest = The Mini-Balance Evaluation Systems Test, MVPA = Moderate-to-vigorous intensity physical activity, LIPA = Light-intensity physical activity, SB = Sedentary behaviours, ilr_1_ = isometric log-ratio coordinate number 1, HADS = Hospital Anxiety and Depression Scale, TMT IV = Trail Making Test IV, S-ESES = Self-efficacy for exercise, ABC = Activities-specific balance confidence. Continuous variables are presented as mean ± SD and categorical variables as n (%).

**Figure 1.**
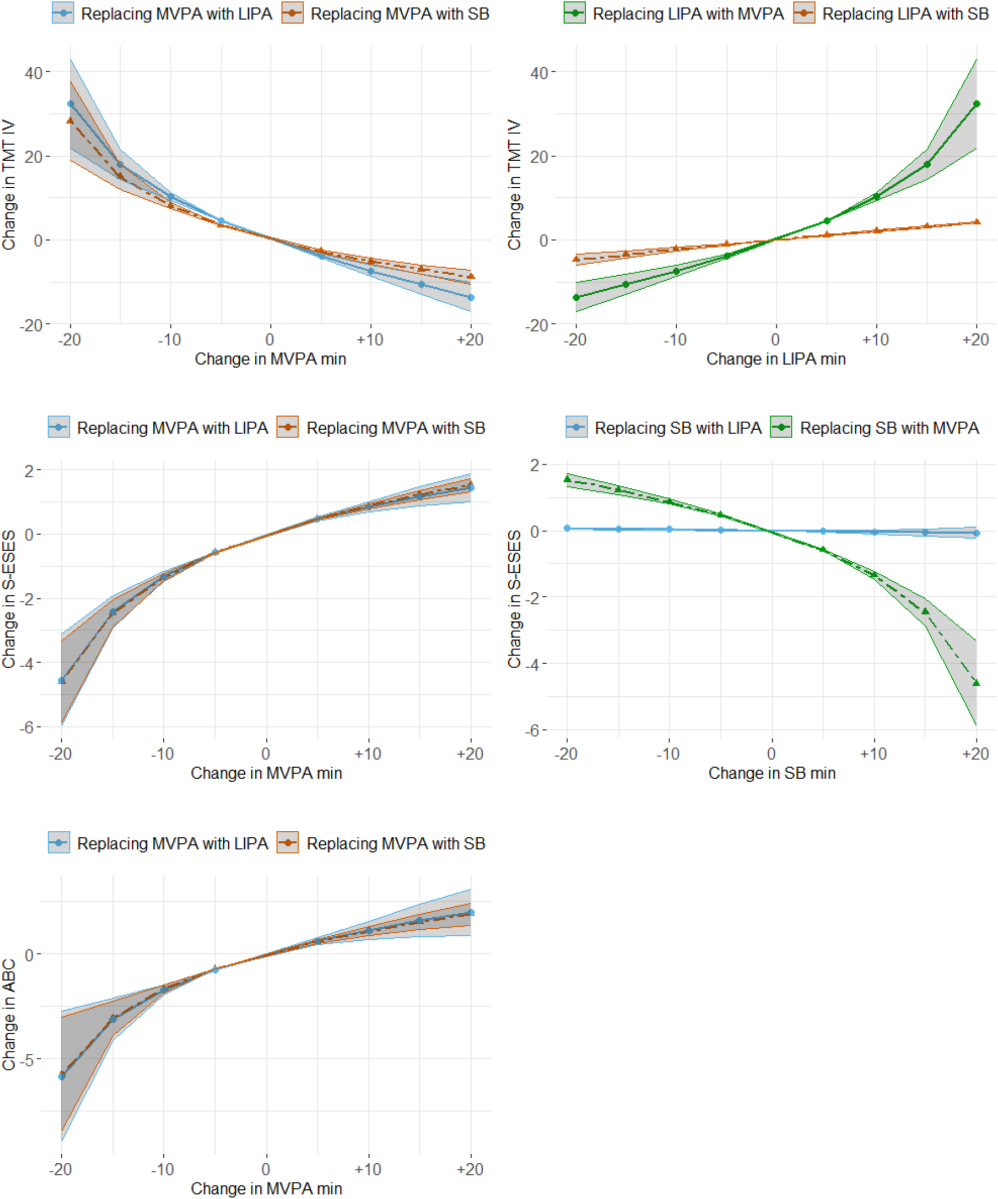
Estimated differences and confidence intervals in the outcomes with reallocation of minutes spent in MVPA, LIPA or SB. Analyses were adjusted for the covariates age, gender, and disease duration. MVPA = Moderate-to-vigorous intensity physical activity, LIPA = Light-intensity physical activity, SB = Sedentary behaviours, TMT IV = Trail Making Test IV, S-ESES = Self-efficacy for exercise, ABC = Activities-specific balance confidence.

Table 2 shows results from the linear regression analysis between each outcome and the relative time spent in each movement behaviour. No associations were found between relative time spent in any movement behaviour and depression or anxiety. More relative time in MVPA and less relative time in LIPA were associated with higher levels of executive functions (TMT IV completion time). More relative time in MVPA and less relative time in SB was associated with higher self-efficacy for exercise (S-ESES). More relative time in MVPA was associated with higher balance confidence (ABC). Figure 1 and Appendix 1 provide additional details of how associations change when, in this theoretical model, time was reallocated between different movement behaviours. An increase in relative time in MVPA was associated with increased executive function (reductions in TMT IV completion time), and a decrease in relative time in MVPA was associated with a decreased executive function. The associations were similar both when time was replaced with LIPA or SB. However, a better executive function was observed when relative time in MVPA was replaced with LIPA, compared to SB. Replacing relative time in LIPA with SB slightly improved executive function.

**Table 2.** Linear regression associations between non-motor characteristics and relative time in each movement behaviour.

	MVPA		LIPA		SB	
	**Crude model**	**Adjusted model**	**Crude model**	**Adjusted model**	**Crude model**	**Adjusted model**
**HADS Depression**	−0.44 (−0.98 to 0.11)	−0.56 (−1.15 to 0.03)	0.18 (−0.92 to 1.28)	0.32 (−0.83 to 1.46)	0.26 (−0.67 to 1.19)	0.25 (−0.72 to 1.21)
**HADS Anxiety**	−0.12 (−0.88 to 0.64)	−0.02 (−0.84 to 0.81)	0.79 (−0.75 to 2.33)	0.39 (−1.21 to 1.98)	−0.67 (−1.97 to 0.63)	−0.37 (−1.72 to 0.97)
**TMT IV**	−25.00 (−36.82 to −13.18)*	−18.09 (−30.01 to −6.17)*	28.22 (3.73 to 52.72)*	29.87 (5.91 to 53.83)*	−3.22 (−23.37 to 16.92)	−11.78 (−31.46 to 7.90)
**S-ESES**	3.31 (1.85 to 4.77)*	2.88 (1.31 to 4.45)*	−0.14 (−3.11 to 2.83)	−0.03 (−3.11 to 3.04)	−3.17 (−5.65 to −0.70)*	−2.84 (−5.42 to −0.27)*
**ABC**	5.06 (1.56 to 8.56)*	3.61 (0.02 to 7.19)*	−4.47 (−11.85 to 2.92)	−1.11 (−8.44 to 6.23)	−0.59 (−6.67 to 5.49)	−2.50 (−8.50 to 3.51)

Table shows the beta coefficients and CI for the first ilr coordinate (describing time spent in a specific behaviour relative to time in the remaining behaviours). Adjusted model includes covariates age, sex, and disease duration. MVPA = Moderate-to-vigorous intensity physical activity, LIPA = Light-intensity physical activity, SB = Sedentary behaviours, HADS = Hospital Anxiety and Depression Scale, TMT IV = Trail Making Test IV, S-ESES = Self-efficacy for exercise, ABC = Activities-specific balance confidence.

*Significant association with the outcome.

In this theoretical model, an increase in relative time in MVPA was associated with increased self-efficacy for exercise and a decrease in relative time in MVPA was associated with a decrease in self-efficacy for exercise. However, the decrease in relative time in MVPA contributed to a greater decrease in self-efficacy for exercise, than gains observed when relative time in MVPA was increased. These relationships were similar whether the reallocated time came to or from LIPA or SB. Reallocating relative time to or from SB and replacing it with LIPA did not affect self-efficacy for exercise.

Reallocating relative time to MVPA from LIPA or SB was associated with increased balance confidence, and reallocating relative time from MVPA to LIPA or SB was associated with decreased balance confidence. Decreased relative time in MVPA contributed to a greater decrease in balance confidence compared to the gains observed by increasing the relative time in MVPA.

## Discussion

This study aimed to investigate associations between relative time in the movement behaviours MVPA, LIPA and SB, and non-motor characteristics in people with PD, and to investigate the theoretical changes which occur in non-motor characteristics when time in different movement behaviours was reallocated.

### Associations between movement behaviours and non-motor characteristics

Our findings show that more relative time spent in MVPA was associated with higher levels of executive function. Additionally, poorer executive function was associated with more time spent in LIPA. This is consistent with another cross-sectional study among people with PD, which found that absolute time in MVPA, but not in LIPA, was associated with higher levels of executive function.^31^ However, movement behaviours were analysed as absolute time, and therefore do not account for their compositional nature.^31^ It is known that executive function is critical for engaging in healthy movement behaviours, and impaired executive function directly affects motor performance, especially gait and balance.^32^ These cognitive challenges increase the risk of falls and limit movement which, in turn, worsens both motor and non-motor symptoms. Studies with cross-sectional designs cannot, however, make claims about the directionality of associations, and a bi-directional link between MVPA and cognition is plausible. Evidence from experimental studies support such associations, showing positive effects of cognitive training on physical activity levels.^33^ Notably, positive effects of higher movement intensities (65% of heart rate reserve) on executive function have been reported among older adults.^34^

The current study found associations between higher self-efficacy for exercise and more relative time spent in MVPA, as well as associations between lower self-efficacy for exercise and more relative time spent in SB. Previous cross-sectional studies in PD have reported similar associations, both when physical activity was self-reported^35^ and measured by activity monitors.^10^ These findings add to the evidence base concerning exercise self-efficacy in PD and its relative importance for time spent in higher intensity physical activity. Higher levels of self-efficacy might foster motivation to commence and maintain exercise programs, even when facing PD-related challenges like fatigue, rigidity, or tremor.^36,37^ People with PD often face physical and psychological barriers to exercise, such as fear of falling or perceived physical limitations.^36^ Heighted self-efficacy may empower people to overcome these obstacles and engage in exercise. However, as with cognition, this relationship may also be bi-directional. A person with greater exercise self-efficacy may be more motivated to engage in higher-intensity physical activity, while increased self-efficacy for exercise may also develop following more intense physical activity engagement. Gaining experience through physical activity can serve as a catalyst for further motivation.^38^ Evidence from intervention studies have yielded mixed results. While improvements in exercise self-efficacy in PD have been reported,^39^ a systematic review found insufficient evidence to support this conclusion.^40^

Our findings demonstrate associations between higher balance confidence and a greater relative amount of time spent in MVPA, whereas no such associations were observed for relative time spent in LIPA. This result aligns with a previous cross-sectional study in individuals with PD that reported higher perceived balance confidence to be associated with more time engaged in leisure time physical activity, as assessed by the Physical Activity Scale for the Elderly (PASE) leisure score.^11^ Similarly, a study conducted among community-dwelling older adults found that higher perceived balance confidence correlated with more time spent in MVPA (measured as absolute time), but no such correlation was identified for LIPA.^41^ Together, these findings suggest that physical activity intensity needs to reach at least the MVPA level to positively influence balance confidence, and that LIPA alone may be insufficient. Enhancing balance confidence is particularly important for individuals with PD and is also shown appears to influence fall risk.^42^

Our study found no associations between anxiety or depression and relative time in any movement behaviour. However, as shown in Table 1, mean HADS scores for both anxiety and depression were below 11, indicating that the sample was not particularly anxious or depressed.^43^

### Theoretical reallocation of time between movement behaviours

A strength of the isotemporal substitution analyses is the provision of theoretical changes in symptoms with altered movement times. Regarding cognition, a theoretical reallocation of 20 min from SB to MVPA would result in a 9 s improvement in completion time of the executive function test. Conversely, if 20 min of MVPA was replaced from with SB, the completion time would be worsened by 28 s (see details in Appendix 1). Notably, the effect was slightly stronger when replacing MVPA with LIPA than SB. This result is somewhat counterintuitive, and additional work will be necessary to confirm the voracity of this finding. Regardless, these results indicate that to theoretically improve executive function in the most optimal way, individually tailored rehabilitation could focus on supporting people with PD to increase their movement intensity from low to moderate-vigorous or, as noted below, maintain existing MVPA. A personalised dialogue may help identify appropriate activity types and intensity levels,^44^ while also considering ecological factors—such as social and physical environments—that influence movement behaviour.^45^ For both self-efficacy for exercise and balance confidence, reallocating time to MVPA resulted in the strongest theoretical effects on these non-motor outcomes, regardless of whether time was reallocated from SB or LIPA. Although these outcomes measure separate constructs, similarities in the trend lines may reflect how these behavioural characteristics are related. Self-efficacy relates to one's beliefs in their capabilities to achieve specific tasks and contributes to greater confidence in one's abilities and decisions.^9^ Such increases in confidence could extend to balance specific confidence. The increases in confidence (both in regard to self-efficacy and balance), could lead to increased participation. Further, higher levels of balance confidence in PD have been associated with a lower fall risk.^46^ Therefore, improvements in balance confidence related to increased MVPA (71 to 77 with 20 min of increase in MVPA, see appendix 1) could positively impact fall-risk.

For all outcomes, similar trends indicate that the negative impact which occurs upon loosing 20 min of MVPA is relatively larger than the positive impact that occurs when the same time is gained. In other words, assuming individuals are currently participating in MVPA, potential loss of this type of exercise is greater than potential gain, when similar time periods are reallocated at higher movement intensity. These results add strength to an important clinical message, although increasing time in MVPA is to be preferred, maintaining current MVPA levels is of major importance. The trend lines between MVPA and executive function, self-efficacy for exercise and balance confidence follows the same pattern as the dose-response relationship between physical activity and health from World Health Organization physical activity recommendations.^47^ Altogether, this highlights the importance of trying to reach the most inactive individuals where the greatest benefits can be achieved when increasing time spent in physical activity at higher intensities, such as MVPA.

### Strengths and limitations

Inclusion criteria for this study were shaped by safety considerations when performing an unsupervised home exercise intervention, therefore people who walked unaided indoors are overrepresented. Participants included may not be representative of the general Parkinson population due to high levels of MVPA and low levels of anxiety or depression. Moreover, the distribution of MVPA (as seen in Table 1) indicates that a small proportion of the participants were more active than the group mean. This will affect the external validity of the results and the applicability to the general Parkinson population. Nonetheless, this sample is likely to reflect individuals with Parkinson's disease who are inclined to seek physiotherapy interventions and are receptive to lifestyle modifications, including physical activity-based interventions, within the context of primary care. To our knowledge, previous studies exploring the associations between movement behaviours and non-motor characteristics in people with PD have not addressed the compositional nature of movement behaviours as relative to each other. More traditional methods such as analysis of the absolute time of each movement behaviour, measured by accelerometers^13^ or self-reported data, has been used.^48^ A main strength of this study is the use of accelerometers to capture movement behaviour data. However, one limitation is that the accelerometers were not worn during the night which exclude the possibility to measure the complete 24 h movement patterns. Another limitation is the cross-sectional design of the study, meaning that we cannot claim any causal inference or rule out reversed causality.

## Conclusion

In people with PD, more relative time spent in MVPA was associated with higher levels of executive function, stronger self-efficacy for exercise and better perceived balance confidence. Theoretical reallocation of time showed that loosing 20 min of MVPA had a stronger negative impact on the outcomes compared to the theoretical benefits that were gained by increasing MVPA by 20 min. These findings can be adopted in clinical settings by communicating the importance of maintaining time in MVPA to people with PD.

## Appendix 1 Predicted outcomes and 95% CI for theoretically reallocated steps of 5 min between the movement behaviours (one-for-one reallocations).

**Table:**

	TMT IV				S-ESES				ABC	
**Time reallocated**	**MVPA to/from SB**	**MVPA to/from LIPA**	**LIPA to/from MVPA**	**LIPA to/from SB**	**MVPA to/from SB**	**MVPA to/from LIPA**	**SB to/from MVPA**	**SB to/from LIPA**	**MVPA to/from SB**	**MVPA to/from LIPA**
**−20 min**	146.2 (124.3 to 168.1)	150.3 (127.1 to 173.5)	104.3 (88.2 to 120.4)	113.1 (99.2 to 127.0)	23.3 (20.4 to 26.2)	23.4 (20.3 to 26.5)	29.5 (27.6 to 31.3)	28.0 (26.4 to 29.6)	71.0 (64.4 to 77.6)	70.9 (64.0 to 77.9)
**−15 min**	132.8 (117.3 to 148.4)	136.0 (119.8 to 152.1)	107.3 (92.3 to 122.2)	114.4 (101.0 to 127.8)	25.5 (23.4 to 27.5)	25.5 (23.4 to 27.7)	29.2 (27.4 to 30.9)	28.0 (26.4 to 29.6)	73.7 (69.0 to 78.4)	73.6 (68.8 to 78.5)
**−10 min**	126.0 (112.7 to 139.4)	128.1 (114.6 to 141.6)	110.5 (96.6 to 124.4)	115.6 (102.6 to 128.7)	26.6 (24.8 to 28.3)	26.6 (24.8 to 28.4)	28.8 (27.1 to 30.5)	28.0 (26.4 to 29.6)	75.1 (71.0 to 79.2)	75.1 (71.0 to 79.2)
**−5 min**	121.4 (108.8 to 134.0)	122.5 (109.9 to 135.0)	114.0 (100.9 to 127.1)	116.8 (104.0 to 129.6)	27.4 (25.7 to 29.0)	27.4 (25.7 to 29.0)	28.4 (26.7 to 30.1)	28.0 (26.3 to 29.6)	76.0 (72.2 to 79.9)	76 (72.2 to 79.9)
**0 min (the predicted mean of the composition)**	117.9 (105.3 to 130.5)	117.9 (105.3 to 130.5)	117.9 (105.3 to 130.5)	117.9 (105.3 to 130.5)	27.9 (26.3 to 29.6)	27.9 (26.3 to 29.6)	27.9 (26.3 to 29.6)	27.9 (26.3 to 29.6)	76.8 (73.0 to 80.6)	76.8 (73.0 to 80.6)
**+5 min**	115.1 (102.3 to 127.9)	114.0 (100.9 to 127.1)	122.5 (109.9 to 135)	119.0 (106.6 to 131.4)	28.4 (26.7 to 30.1)	28.4 (26.7 to 30.1)	27.4 (25.7 to 29.0)	28.0 (26.3 to 29.6)	77.4 (73.4 to 81.3)	77.4 (73.4 to 81.4)
**+10 min**	112.8 (99.6 to 126.0)	110.5 (96.6 to 124.4)	128.1 (114.6 to 141.6)	120.0 (107.7 to 132.4)	28.8 (27.1 to 30.5)	28.8 (27.0 to 30.6)	26.6 (24.8 to 28.3)	27.9 (26.2 to 29.6)	77.9 (73.8 to 81.9)	77.9 (73.6 to 82.2)
**+15 min**	110.8 (97.1 to 124.5)	107.3 (92.3 to 122.2)	136.0 (119.8 to 152.1)	121.0 (108.7 to 133.4)	29.2 (27.4 to 30.9)	29.1 (27.2 to 31.0)	25.5 (23.4 to 27.5)	27.9 (26.1 to 29.6)	78.3 (74.1 to 82.5)	78.4 (73.7 to 83.0)
**+20 min**	109.1 (94.8 to 123.3)	104.3 (88.2 to 120.4)	150.3 (127.1 to 173.5)	122.0 (109.6 to 134.4)	29.5 (27.6 to 31.3)	29.4 (27.3 to 31.5)	23.3 (20.4 to 26.2)	27.9 (26.1 to 29.7)	78.7 (74.3 to 83.0)	78.8 (73.8 to 83.7)

The models include covariates age, gender, and disease duration. MVPA = Moderate-to-vigorous intensity physical activity, LIPA = Light-intensity physical activity, SB = Sedentary behaviours, TMT IV = Trail Making Test IV, S-ESES = Self-efficacy for exercise, ABC = Activities-specific balance confidence.
